# Positive postpartum depression screening practices and subsequent mental health treatment for low-income women in Western countries: a systematic literature review

**DOI:** 10.1186/s40985-017-0050-y

**Published:** 2017-01-31

**Authors:** Elinor Hansotte, Shirley I. Payne, Suzanne M. Babich

**Affiliations:** 1 Department of Health Policy and Management, Indiana University Richard M Fairbanks School of Public Health at IUPUI, 1050 Wishard Blvd, Indianapolis, IN 46202 USA; 2 Department of Applied Health Science, Indiana University School of Public Health, 1025 E. 7th Street, Suite 111, Bloomington, IN 47405 USA

**Keywords:** Postpartum depression, Postpartum depression screening, Postpartum depression treatment, Mental health, Women’s health

## Abstract

**Problem statement and significance:**

Left undiagnosed and/or untreated, the short-and long-term sequelae of postpartum depression may negatively impact both mother and child. In Western countries, access to mental health care is influenced by socioeconomic factors. The objective of this systematic literature review is to compile factors that hinder and improve access to postpartum depression treatment in low-income women after a positive screen for postpartum depression. The key question of focus is: what are the characteristics associated with access to mental health treatment for low-income women with a positive postpartum depression screen in Western countries?

**Methods:**

A PRISMA-based systematic literature review was conducted of studies published in English before February 2016 that looked at treatment for postpartum depression in low-income women who had been identified with the condition. PubMed and EBSCO databases were searched using MESH and key terms and found 100 articles that met the selection criteria. After review by two independent researchers, 18 studies with 17 unique populations were included in the literature review.

**Results:**

Two independent abstractors searched the included articles for themes surrounding impediments and advantages for low-income women identified with postpartum depression in obtaining mental health treatment. Characteristics of successful mental health treatment included studies that employed the use of a home visitor and those that separated outcomes for women with previous mental health treatment. Themes that emerged as treatment obstacles included cultural barriers, physical barriers, systemic health care barriers, and social barriers.

**Implications for practice:**

This review will help to better inform screening and treatment priorities for those in the medical field who may encounter women experiencing postpartum depression and are not aware of the various barriers to care specific to low-income women. This review will also help policymakers identify specific obstacles that are not addressed in postpartum screening mandate policies which can affect the implementation of these policies.

## Background

Women are at risk to develop depression after childbirth, also known as the postpartum period [1]. Postpartum depression (PPD), defined as nonpsychotic depression occurring up to 1 year after childbirth, is a “crippling mood disorder that erodes away at the joy and happiness of new mothers” [2–4]. PPD is recognized as an international public health concern. There is an estimated prevalence of PPD of 10–20% among mothers in Western countries [5–7]. The few studies that have examined PPD prevalence in developing countries show similar or higher prevalence of this condition [5, 6, 8, 9]. If undiagnosed and/or untreated, PPD can adversely affect the mother and child, as well as the mother-child relationship [3]. For the mother, PPD has a deleterious effect on all social and familial relationships, functional status (including impaired parenting behaviors), and ability to care for the infant and herself, including increasing the risk of the mother harming herself. Maternal suicides account for up to 20% of all postpartum deaths [10, 11]. The infant is at increased risk for long-term health and developmental problems (including cognitive, language, and school delays) and behavioral issues [1, 2, 4, 12].

Although the exact etiology of PPD has not been determined, there are known risk factors for developing this condition [13]. Women with lower socioeconomic status or educational attainment, history of depression prior to pregnancy, lack of social support, history of substance use/abuse, previous birth(s), and lower employment status are at higher risk for developing PPD [1, 2, 7, 14]. Studies have shown that PPD occurs two to four times as often for women living in poverty, as compared to middle-or-upper-income women; socioeconomic status is often thought to be the most consistent predictor of PPD [1, 2, 4, 7, 11, 12, 15]. In the USA, the population most at risk for PPD is disproportionately represented by racial/ethnic minorities, such as African-American and Hispanic women. Perhaps relatedly, these are the populations affected the most by health care inequities associated with the lack of resources characteristic of low socioeconomic status [11, 16].

The most significant factor in the duration of PPD is the time it takes for the new mother to receive adequate, individualized treatment in the form of pharmacological, psychotherapeutic, and/or social support intervention [3, 10, 12, 17]. Although it does not guarantee treatment, the woman must first be screened for PPD, which is often accomplished using a tool such as the Edinburgh Postnatal Depression Scale (EPDS) or the Postpartum Depression Screening Scale or a self-report used as a proxy for clinical assessment [4, 14, 16, 18]. Routine screening is encouraged, but not mandated, in most Western countries, but it is estimated that only about half of women in these countries with PPD receive any type of evaluation or treatment for the condition [6, 7, 11, 14]. Low-income women have lower rates of screening and treatment for PPD, in part due to lack of knowledge about PPD, economic barriers, and stigma [14].

The objective of this systematic literature review is to summarize the practices used to screen for PPD, as well as assess barriers to mental health treatment after a positive screen is received, among low-income women. The key question we want to address is: what are the characteristics associated with access to mental health treatment for low-income women with a positive postpartum depression screen in Western countries? This review initially looked at literature with no geographic boundaries, but only studies from Western countries fit the inclusion criteria. For this review, PPD symptoms are treated as either a clinical diagnosis of postpartum depression or a self-diagnosis of PPD by study participants. Studies included in the review will be examined to determine how authors established the definition of a positive PPD screen and the rate and process by which women were referred for and sought treatment. Perceived barriers to treatment will be extracted from the articles to gain a broader understanding of the obstacles to mental health treatment for low-income postpartum women with PPD. The review targets low-income women, as this population universally has more barriers to care and thus is more vulnerable than women with greater access to resources.

### Global history of PPD

A brief historical overview is provided for context to help readers better understand the nature of the problem today.

Mood disturbances and psychoses during the postpartum period have been described since at least the time of Hippocrates, who considered the illnesses to originate specifically within the puerperal and lactation periods [19, 20]. The first published paper devoted specifically to puerperal mental illness was printed in 1858 by Louis-Victor Marcé. Marcé’s *Treatise on Insanity in Pregnant, Postpartum and Lactating Women* found that although the symptoms that pregnant and postpartum women were experiencing could be found in other mental disturbances, the combination of symptoms was distinct to the functional changes occurring within the reproductive system after childbirth and should therefore be classified as a separate diagnosis [21].

However, there was no consensus on the definition of depression in postpartum women until the late twentieth century. In the mid-1900s, Britain differentiated postpartum psychiatric disorders from nonpuerperal mental illness, but the USA viewed PPD either as harmless, fleeting “baby blues,” or as affective or schizophrenic episodes [21, 22]. Seeking a universal definition, clinicians and researchers began advocating for a clinical diagnosis of PPD in order for the condition to be recognized by health care systems [23]. In the USA, PPD was formally recognized in 1994 as a clinical diagnosis in the Fourth Edition of the *Diagnostic and Statistical Manual of Mental Disorders* (DSM) to validate the stress some new mothers experience during the postpartum period [13]. Outside of the DSM, in Western countries, there are currently three accepted types of postpartum mood disorders: postpartum blues, the most common postpartum mood syndrome, defined as a mild and short-lasting ailment; PPD, an episode of major or minor depressive disorder in the postpartum period; and postpartum psychosis, a rare, acute psychiatric episode in the postpartum period [18, 21].

Integrating mental health screening into primary care for pregnant and postpartum women and providing follow-up and treatment is a growing concern worldwide [18]. Australia and New Zealand have published national recommendations stating that it is the provider’s responsibility to be aware of the risks of PPD, to be able to identify the condition and to make the appropriate referrals for mental health treatment. Similarly, the Norwegian government is endorsing initiatives targeted at mental health issues in women during and after pregnancy [11]. In the USA, the Patient Protection and Affordable Care Act (ACA) went into effect in 2010 and contains language for providing support services to women and research support for PPD. Even before the ACA, individual states such as New Jersey (2006) and Illinois (2008) took action towards mandating screening or physician reimbursement for screening for PPD [18]. When examining strategies to better incorporate mental health care in developing countries, the World Health Organization found that the use of short validated screening tools has been effective in detecting patients with issues like PPD. In a study performed in Nigeria, the EPDS was an effective tool in identifying women with PPD [5].

Much of the research about PPD (including screening and treatment) has been confined to Western countries. Although the physiology of human pregnancy is the same worldwide, the mother’s experience is greatly impacted by cultural factors. Some cultures do not recognize PPD, and mothers are sometimes reluctant to admit symptoms due to cultural expectations of women’s behavior in motherhood. Since PPD is not universally recognized and treated, more research is needed across cultures to aid in a fuller understanding of the burden of PPD globally [20].

## Methods

A systematic literature review was conducted to identify postpartum depression screening practices and mental health treatment for low-income women.

### Data sources and search strategy

The Preferred Reporting Items for Systematic Reviews and Meta-Analyses (PRISMA) guidelines were used as a basis for our methodology. PubMed and EBSCO databases (no specified beginning date through February 2016) were comprehensively searched using variations and combinations of the following MESH and key terms and phrases: “pregnancy” and “pregnancy” [MESH]; “female” [MESH]; “Medicaid” [MESH]; “poverty” [MESH]; “medical assistance” [MESH]; “depression, postpartum” [MESH]; “depression” and “depression” [MESH]; “depressive disorder” [MESH]; “health services accessibility” [MESH]; “mental health services” [MESH]; “diagnosis” [MESH]; “screening”; “maternal depression and poverty”; “postpartum depression and poverty”; “maternal depression in low-income women”; “maternal depression”; and/or “postpartum depression in poor women”.

### Inclusion and exclusion criteria

The inclusion and exclusion criteria for this systematic literature review were structured with the intent of understanding the heretofore-not-compiled topic of barriers to treatment specific to low-income women with a positive postpartum depression screen. Only empirical, English-language, peer-reviewed publications were considered for this study. In order to focus on scholarly evidence, we excluded reviews (including analyses), case reports, letters to the editor, executive summaries, governmental reports, and commentaries. We also excluded articles that did not include treatment as an outcome, articles that did not look at low-income women specifically, incomplete studies, articles that did not have a postpartum women group, and those for which treatment as an outcome was theoretical rather than measured. The term low-income used in this systematic literature review is not based on one specific definition; articles that self-identified low-income populations were considered for inclusion. Included studies identified low-income women with the following measures: WIC recipients, women below the federal poverty rate, income categories with low-income specifications, Early Head Start participants, Medicaid (and state-specific Medicaid program) recipients, women on public assistance, women who used public aid clinics, and food stamp recipients.

### Data extraction

To review data, an extraction form was designed by the researchers based on the outcomes of the study. This included author(s), sample size, study design, study location by country, participant demographic information, tool used to determine PPD, how treatment was assessed, and presence of a comparison group. Disputes on article categorization during the article evaluation process between two raters were minimized through the employment of the extraction form.

## Results

### Evaluation of studies

**Fig. 1:**
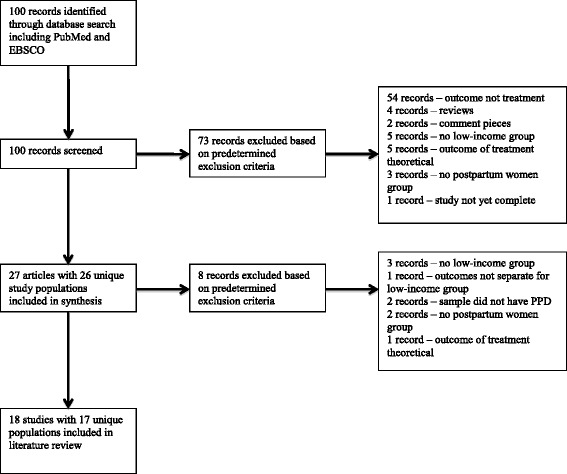
PRISMA diagram of eligibility requirements

Our initial search identified 100 articles (see Fig. 1). The titles and abstracts of these articles (or full articles when an abstract was not available) were independently reviewed by two authors. Any disagreements were resolved through a consensus-building joint review of the abstract or complete article. There were no duplicate articles. We also went through the reference lists of included studies and found two new articles for inclusion in this review. After review, 27 articles were included in the literature review. Additionally, 54 papers were excluded because the outcome was not related to treatment, four papers were excluded because they were reviews, two papers were excluded because they were commentaries, five papers were excluded because low-income women were not studied, five papers were excluded because the outcome of treatment was theoretical, three papers were excluded because the sample was not postpartum women, and one paper was excluded because the study had not yet been completed.

After screening the 27 articles included in the literature review, eight were excluded based on predetermined criteria. Of those, three papers were excluded because there was no low-income group, one paper was excluded because the outcomes were not separated for the low-income group, two papers were excluded because the sample did not have PPD, two were excluded because the sample was not postpartum women, and one was excluded because the outcome of treatment was theoretical.

### Analysis

#### Study population characteristics of 18 included studies

Using the qualitative analysis technique of constant comparative analysis, data extracted from included articles were inductively coded by treatment characteristics and barriers. Patterns and themes that were developed from the coded data centered on two major topics: characteristics of PPD treatment and barriers to mental health treatment, which have been outlined through subheadings below. Table 1 outlines the characteristics of each article. Studies varied greatly by study design, assessment tool to determine PPD, and treatment type. Four studies were randomized controlled studies and thus provided the highest level of evidence. Six different standardized screen tools, three different diagnostic codes, self-report, or a primary care evaluation were all methods used to indicate a positive PPD screen. Some studies provided treatment for participants, and others asked if treatment was sought.

**Table 1:** Characteristics of included articles

Author (year)	Study design (sample size)	Country	Participant characteristics		Postpartum depression		Treatment		
			Race/ethnicity distribution	Mean age	Measure	Definition	Assessment	Type	Treatment outcome
Abrams et al. (2009) [28]	Cohort (14)	USA	B—43%, L—57%	27	Self-report	Self-report of PPD symptoms	Self-assessment through focus groups, individual interviews	No women in this study sought treatment	No women in this study sought treatment
Beeber et al. (2004) [15]	RCT (16)	USA	W—38%, B—62%	26.6	CES-D	16 or higher on CES-D	Intervention group	Therapeutic relationship with master-prepared psychiatric mental health nurse home visitor —8 face-to-face contacts over 8–10 weeks, then about 5 phone calls per week for 8 weeks	50% randomized to treatment intervention, no one in control group (*n* = 8) sought treatment during study period
Bobo et al. (2014) [34]	RCT (2343)	USA	W—65%, B—15%, A—1%, U—19%	26.4	EPDS, PHQ-9	10 or more on EPDS or PHQ-9	Intervention group	Nursing staff: follow-up telephone calls dealing with antidepressant drug therapy or counseling initiation, adherence, and adverse effects	Treatment selection at discretion of participant (not reported); of 1246 who completed supplemental survey, 9.2% were unable to access treatment
Broom et al. (2015) [30]	Cohort (58)	USA	W—14%, B—83%, M—3%	NR	EPDS, BDI-II	10 or more on EPDS	All study participants received treatment	Supportive 4 text messages/week for 6 months; CBT counseling—unlimited access to therapeutic services and telephone support	CBT use not reported; 1.9% of total texts sent requested a call back from researchers
Callister et al. (2011) [35]	Cohort (20)	USA	L—100%	24	PDSS-Spanish	Cutoff score of 60 on PDSS for positive screen	Interview	Interview to assess PPD thoughts, treatments, and barriers	Women were not directly asked about their own treatment
Chaundron et al. (2005) [27]	Cohort (218)	USA	L—100%	26.5	EPDS	Self-recognition: “yes” to question, “have you thought that you needed help with sadness or depression”; 10 or more on EPDS	Self-report	Referral to mental health specialist after self-report to physician	28% of participants felt they needed help, 47.5% of those participants spoke to a provider, and 33% of those were referred to a mental health specialist
Crockett et al. (2008) [32]	RCT (36)	USA	B—100%	23.4	CSQ, EPDS	27 or more on CSQ, 10 or more on EPDS	Intervention group	Four 90-min weekly group sessions and a 50-min individual booster session 2 weeks after delivery	52.7% participants randomized to treatment
Geier et al. (2015) [36]	Retrospective cohort (6030)	USA	W—9%, B—3%, A—1%, L—85%, O—1%, U—1%	NR	ICD-9 code	Any ICD-9 code for depressive illness as primary or secondary diagnosis	ICD-9 codes for treatment	(1) Received antidepressant medication only, (2) received psychotherapy only, (3) received both antidepressant medication and therapy, and (4) received neither	4.1% received antidepressants postpartum; 56% of depressed cohort received treatment vs 74% of control group
Kozhimannil et al. (2011a) [40]	Cohort (30,955)	USA	W—42%, B—43%, O—15%	NR	New Jersey’s Medicaid data	No documented diagnosis to indicate treatment initiation	Medicaid data on filling a prescription for antidepressant medication or having had an outpatient mental health visit in 6 months after delivery	Antidepressants or outpatient mental health visit	7% initiated mental health treatment, of which 90% used outpatient mental health services and antidepressants
Kozhimannil et al. (2011b) [39]	Retrospective cohort (29,601)	USA	W—44%, B—45%, A—11%	NR	ICD-9 code	Any ICD-9 code for depressive illness as primary or secondary diagnosis	ICD-9 codes for treatment	Filling a prescription for antidepressant medication or having had an outpatient mental health visit in 6 months after delivery	9% white women, 4% black women, and 5% Latina women initiated treatment
Letourneau et al. (2007) [25]	Cohort (41)	Canada	W—81%, N—10%, O—9%	31.27	EPDS	10 or more on EPDS	Interview	Support seeking, support needs, barriers to support, and preferences for support intervention	43.9% were unable to locate support programs for PPD; those with treatment not reported
Logsdon et al. (2009) [29]	Cohort (9)	USA	W—22%, B—44%, L—22%, N—11%	16	Self-report	Self-report of PPD symptoms	Semi-structured interview: What actions might you take if you had a lot of negative (sad/unhappy) feelings after having a baby? Probes: Is there anything you can do? What might you do first? What do you think is the best way to handle a situation like this? Who would be someone you could go to help you? Anyone else? Have you had any experience in getting help in a situation like this? If so, what were the challenges to getting help with negative feelings after having a baby?	No women in this study sought treatment	Participants were asked if they had received help for negative feelings, but outcome is not reported in paper
McGarry et al. (2009) [26]	Retrospective cohort (213)	USA	W—85%, O—15%	NR	Primary Care Evaluation of Mental Disorders Patient Health Questionnaire	Response of “always” or “often” to the following: (1) Since your new baby was born, how often have you felt down, depressed, or hopeless? and (2) Since your new baby was born, how often have you had little interest or little pleasure in doing things?	Survey question: “Since your new baby was born, did you seek help for depression from a doctor, nurse, or other health care worker?”	Type of treatment not specified	60.5% (unweighted) women who reported PPD did not seek help
O’Mahoney et al. (2012) [37]; O’Mahoney et al. (2013) [38]	Cohort (30)	Canada	B—7%, A—50%, L—43%	NR	EPDS	10 or more on EPDS	Self-report in interview	Type of treatment not specified	Patents were unfamiliar with or unaware of treatment options
Price et al. (2009) [31]	Cohort (1086)	USA	W—70%, B—27%, L—2%, O—1%	24.6	PHQ	Published guidelines for PHQ	Self-report on questionnaire	Type of treatment not specified	Treatment specific to postpartum women not reported: 38% sample with depressive symptoms, 3.4% postpartum, 2.3% current psychiatric medication use, and 5.5% current counseling
Song et al. (2004) [33]	Cohort (3841)	USA	W—17%, B—83%	24.4	Psychiatric diagnosis	DSM-III-R criteria for severe mental disorders, major depression, minor psychiatric disorders	Medical chard codes for using mental health services	Inpatient, outpatient mental health visits (psychiatrists, psychologists, psychiatric social workers, community psychiatric nurses)	6.4% used mental health services in postpartum period
Surkan et al. (2012) [24]	RCT (679)	USA	L—75%, UTD—25%	26	CES-D	Standard CES-D cut-off	Randomized to control (WIC benefits) or intervention	Five home visits, delivered by paraprofessionals, monthly phone calls from intervention staff	49.6% patients randomized to intervention with treatment

*NR* not reported, *W* white, *B* black or African-American, *L* Latina, *A* Asian, *U* unknown, *M* multiple races, *O* other, *N* native, *UTD* unable to determine, *CES-D* Center for Epidemiologic Studies Depression Scale, *EPDS* Edinburgh Postnatal Depression Scale, *PHQ-9* Patient Health Questionnaire, *BDI-II* Beck Depression Inventory, *PDSS* Postpartum Depression Screening Scale, *ICS-9* International Classification of Diseases, *CSQ* Cognitive Style Questionnaire

#### Characteristics of postpartum depression treatment

Multiple studies found that the use of a home visitor was an effective method of providing some level of PPD care for mothers. Beeber et al. [15] and Surkan et al. [24] used home visitors to either identify or provide care for PPD. Both were randomized controlled studies in which the intervention group with the home visitor showed lower depression scores at follow-up than the control group. The subjects in Letourneau et al. [25] agreed that in-home support would be a preferred treatment method for PPD.

Another theme associated with positive PPD treatment was previous mental health treatment. McGarry et al. [26] and Chaundron et al. [27] found that women who had previously sought mental health treatment were more likely to seek treatment for PPD. Providers and mothers were both better able to recognize symptoms of depression in this population. Abrams et al. [28] and Logsdon et al. [29] found that religion provided comfort for some women through practices like prayer and attending church.

Other topics associated with mothers who sought PPD treatment appeared only in single articles. Broom et al. [30] showed that supportive text messaging, combined with cognitive behavioral therapy, was a way to bridge access barriers in a low-income population. Price and Proctor [31] found that the addition of postpartum screening and treatment in community-based programs such as Healthy Start broadened access for underserved women. While not focusing solely on PPD, Crockett et al. [32] found that targeting social adjustment and role transition for new mothers improved PPD scores. Song et al. [33] found that older women sought treatment more often, due to maturity and a greater awareness about how to find care. Finally, Surkan et al. [24] found a potential link between self-efficacy measures (improved diet, physical activity, and social support) and reduction in PPD symptoms.

#### Barriers to mental health treatment

Cultural barriers to screening and treatment were a major theme across the literature. Stigma, racial barriers, perceptions of motherhood, language barriers, immigration status, religion, and cultural sensitivity were all sub-themes pulled from this broader theme. Mothers in many studies (Abrams et al. [28], Beeber et al. [15], Bobo et al. [34], Broom et al. [30], Callister et al. [35], Letourneau et al. [25], and Geier et al. [36]) did not seek PPD care because of the stigma attached to it. Some reported not wanting to be seen as “crazy” or to have a “real” mental illness. Other women described withdrawing during PPD because they were afraid of the stigma.

One study (Bobo et al. [34]) found that stigma may impact insurance coverage for therapeutic treatments for PPD because of perceptions that PPD is difficult to manage. Along the lines of stigma, perceptions of motherhood affected self-seeking patterns of PPD treatment. Mothers in Abrams et al. [28], Callister et al. [35], and Letourneau et al. [25] had certain ideas about mothers and motherhood. They felt that “good mothers” do not get depressed or that feelings of sadness after having a baby were just part of the motherhood process. Afraid of being seen as a bad mother and having the child(ren) taken from them, many mothers ignored the feelings of PPD. In Logsdon et al. [29], Letourneau et al. [25], and Callister et al. [35], participants attributed PPD feelings to other causes rather than acknowledging PPD.

Similarly, Callister et al. [35] and O’Mahoney et al. [37, 38] examined immigrant and refugee populations and found unique barriers to care, such as an undocumented status, within that subset of low-income women. Geier et al. [36], Kozhimannil et al. [39], and Song et al. [33] discussed racial barriers in PPD care. Overall, white women were more likely than black or Latina women to get a diagnosis of PPD or to get treatment for PPD. Within the Latina population, language barriers may pose a significant barrier to PPD care. Language barriers on the part of the provider and mother that lead to a lack of access for PPD care were identified by Callister et al. [35], Chaundron et al. [27], and O’Mahoney et al. [37, 38]. Abrams et al. [28] and Song et al. [33] found that low-income women, especially minority women, experienced an additional barrier caused by a lack of provider cultural sensitivity. Abrams et al. [28] found that although religion was a comfort to some women, others found it to be a barrier when beliefs encouraged women to accept the way they feel during PPD rather than seek treatment.

Physical barriers to PPD treatment included lack of child care, lack of transportation, lack of financial resources, and housing issues. Abrams et al. [28], Bobo et al. [34], and Callister et al. [35] identified lack of child care to be a barrier to PPD treatment. Abrams et al. [28], Beeber et al. [15], Bobo et al. [34], Callister et al. [35], and Crockett et al. [32] identified lack of transportation as a major barrier to PPD treatment outside the house. Beeber et al. [15] found that even with the convenience of a home visitor, when women did not have a car, they would still miss appointments if a vehicle became suddenly available to them in order to take care of other needs. Women in Crockett et al. [32] specifically discussed a lack of public transportation in rural communities as a barrier to care. Abrams et al. [28], Beeber et al. [15], Callister et al. [35], O’Mahoney et al. [37, 38], and Bobo et al. [34] found financial barriers to be one of the most significant obstacles to PPD treatment. Abrams et al. [28] found that women were not sure if insurance covered mental health treatment so they did not risk the potential expenditure. Women in Callister et al. [35] noted, “if she has no money, how is she going to find help [with PPD]?” and “as Hispanics we do not have insurance and money is what really counts.” Immigrant or refugee women in O’Mahoney et al. [37, 38] were economically dependent on their sponsors, so some of these women were not allowed to get treatment. Finally, Beeber et al. [15] identified housing barriers to PPD treatment. The home visitor found it difficult to focus solely on PPD symptoms when extenuating circumstances, like poor housing, existed.

Different barriers within the health care structure were also discussed and included: insurance issues, access to care, medication concerns, lack of awareness by the mother about PPD itself or treatment options, previous negative experiences with the medical system, and provider error. Bobo et al. [34] and Geier et al. [36] spoke of insurance barriers to PPD treatment. Those without insurance or with inconsistent insurance found it much more difficult, or impossible, to access PPD care. Abrams et al. [28], Broom et al. [30], Letourneau et al. [25], Price and Proctor [31], and O’Mahoney et al. [37, 38] found additional barriers regarding access to PPD treatment. Women did not know where to find treatment, how to get engaged in treatment, or were geographically removed from treatment. O’Mahoney et al. [37, 38] and Song et al. [33] found that women were not aware of PPD in general or the existence of treatment options. Geier et al. [36] found concerns about the safety of antidepressant use while breastfeeding, which was addressed as a barrier to care. Providers were perceived as the cause of two barriers: negative prior experiences by women with PPD and provider error. Abrams et al. [28], Letorneau et al. [25], and O’Mahoney et al. [37, 38] found that women who had previously tried to seek treatment for PPD had been told to wait and see what happened or were given a pharmaceutical intervention without much discussion. This made women feel minimized or dismissed by medical professionals and impacted subsequent treatment for PPD.

O’Mahoney et al. [37, 38] found that women who had had previous health care encounters experienced an unequal balance of power between the provider and patient, which impacted the decision to use the health care system. Geier et al. [36], Chaundron et al. [27], and Letourneau et al. [25] found provider error to be a barrier to treatment for PPD. Geier et al. [36] found that obstetrician/gynecologists felt it to be their responsibility to provide mental health services for PPD, thereby not making a referral to a mental health provider. Chaundron et al. [27] and Letourneau et al. [25] found that providers screened women too early or were not effective in verbally evaluating women because of differences in viewpoints about what constitutes PPD. O’Mahoney et al. [37, 38] found that women also felt that providers were downplaying the symptoms they were experiencing.

Social barriers were the final theme extracted regarding barriers to treatment, with sub-themes of social support, self-help/self-reliance, and policy issues. Abrams et al. [28], Callister et al. [35], Letourneau et al. [25], Logsdon et al. [29], and O’Mahoney et al. [37, 38] found social support barriers to seeking PPD treatment. Callister et al. [35] and Letourneau et al. [25] found that women isolated themselves from others because of PPD. Abrams et al. [28], Callister et al. [35], Logsdon et al. [29], and O’Mahoney et al. [37, 38] found that women often seek help from a social contact rather than seeking professional care. Those without social relationships felt an additional burden when dealing with PPD. Abrams et al. [28], Letourneau et al. [25], and Price and Proctor [31] found that women were more likely to address PPD through self-help/self-reliance than go through a professional channel. One common method for self-help was positive self-talk. Kozhimannil et al. [40] found no increase in PPD treatment from before a law was passed that mandated PPD screening and after the law was passed.

## Discussion

Multiple themes were discussed as possible reasons why low-income women are or are not able to get treatment for PPD after a positive screen. The objective of this literature review is not to address ways to surmount barriers to PPD treatment, as the barriers vary by situation. Rather, the authors focus on answering the key question, which seeks to determine the characteristics associated with access to mental health treatment for low-income women with a positive postpartum depression screen in Western countries. This review provides a compilation of obstacles that low-income women face in accessing treatment for PPD; however, general recommendations are offered in the conclusion. This is also not a critique on screening methods and/or the efficacy of treatment type. The authors do consider certain patterns based on study design choices, like screening tool and treatment type.

Considering the targeted population for this review and the fact that minority populations are more likely to be poor and uninsured [41], it was not surprising that financial barriers were a main reason that women did not seek treatment for PPD. Financial barriers were broader than not merely being able to pay for treatment; women with children could not afford to pay someone to watch the child(ren) during treatment, could not afford transportation to go for treatment, or had insurance issues that impeded getting treatment. Even if a low-income woman had a positive screen for PPD, either through self-recognition of symptoms or a screening tool administered by a clinician, overcoming the next set of barriers was often too difficult. Studies in which a treatment group included home visitors that provided some level of mental health services showed that depression scores were lower for the women with in-home care. Financial barriers may be addressed through home visiting programs because the transportation factor is eliminated. The two studies [15, 24] that showed success through the employment of home visitors as part of the treatment group were randomized controlled studies, providing strong evidence for home visitors as a method of overcoming some barriers low-income women with PPD face.

Stigma was a major barrier to treatment for this population. Low-income women may already feel stigmatized for socioeconomic reasons, and the fear of that stigma being exacerbated by a mental illness may make seeking treatment not seem worthwhile. In addition to reducing financial costs of treatment, home visitation treatment of PPD also increases the opportunity for in-home providers to foster positive social support for the mother among her immediate and extended social circle through education about PPD and addressing misperceptions related to treatment. This includes educating both the mother and members of her support system about the symptoms of PPD, emphasizing that they are not alone in experiencing these symptoms, and explaining that seeking treatment is not a sign of weakness or reflection of a mother’s parenting ability. A woman may also feel freer to talk about her feelings in her home without the fear of being stigmatized.

Many articles reviewed showed that women were not familiar with PPD, did not know how to get treatment for PPD, or thought that PPD was a normal part of motherhood. However, some of these articles did not use standardized methods of assessing PPD, which may minimize the level of evidence for this theme. Cultural perceptions about how a mother should feel and act were major barriers to recognizing that PPD symptoms were an issue. Many women felt that even if they were screened for PPD by a clinician, the issue was not explained in a way that they understood. Many women thought that they could overcome PPD symptoms themselves. These themes surrounding a lack of awareness about PPD and its treatment make it clear that education for providers and patients is vital to normalizing PPD and treatment.

Many women did not want to get treatment for PPD due to negative experiences with mental health or other medical services in the past. Low-income women felt that they were talked down to or dismissed by medical providers; however, previous mental health treatment was seen to be positively associated with treatment for PPD. This is perhaps due to an increased awareness about symptoms. Along the same lines, the literature suggests that physicians must also be aware of the cultural and language differences and barriers that affect low-income women seeking treatment and how these play a role in their health-seeking behavior. This extends beyond using cultural competence as a tool or skill that can be taught and mastered to assist low-income women; rather, there should be a continuous effort by the provider to develop cultural knowledge to provide culturally appropriate treatment. Although none of the included articles for this study directly addressed the confounding role that racial discrimination could play in the differences of treatment for low-income women, specifically women of color, it is important to acknowledge. Kozhimannil et al. [39] found that of the reviewed Medicaid population, white postpartum women were significantly more likely to have an *International Classification of Diseases, Ninth Revision (ICD-9)* code for an outpatient mental health visit or national drug code for antidepressants (i.e., significantly more likely to obtain PPD treatment) than black or Latina women. This indicates that there may be an opportunity to explore this theme in future studies.

When looking at the paper by Kozhimannil et al. [40], in which a mandate for PPD screening did not improve treatment initiation, follow-up, or continued care, the authors suggested that perhaps lawmakers did not consider or address barriers to care in the development of this policy. Studies that looked at specific interventions that focused on some type of community-level support or self-care practices showed improvements in PPD scores. A policy intervention such as that described in the paper by Kozhimannil et al. [40] that only mandates clinicians to complete a checklist that includes a basic PPD screen during postpartum clinical visits may be enhanced when combined with interventions that work to boost the mother’s mental well-being and that also address overcoming barriers to care. In creating strategies to improve access to PPD treatment among low-income women, policymakers need to issue additional guidance on how to best operationalize these policies in ethnically and racially diverse and underserved populations. Creating the policy alone without intentional focus on how it will be implemented is no benefit to those experiencing symptoms of PPD, especially those who are already underserved.

### Limitations

One limitation of this literature review was the publication bias that resulted from the limited number of studies that met the inclusion criteria. Another limitation was the generalizablility of the results or conclusions, as all articles that met the inclusion criteria for this review were conducted in Western countries. Because studies on PPD outside of Western countries did not necessarily look at the same sample population as this review, it is not possible to determine if the low-income definition used for this paper is generalizable to developing countries. It also must be noted that barriers to treatment are not the only indicator of PPD outcomes or symptoms and that not all PPD assessment tools and/or treatments are standardized or equally efficacious.

## Conclusions

Addressing the perceived barriers to mental health treatment for low-income women with PPD symptoms is crucial to increasing access to treatment and receipt of care in this population. Based on the literature examined, there is a need for providers to recognize that formal treatment must take into account the unique cultural experiences of this population and should adjust accordingly to fit the patient’s needs. This may require innovative approaches that move beyond the clinical setting to community-level interventions in order to help dispel negative perceptions regarding PPD and support normalization of the condition. The identification of PPD by a health care worker should be done in a way that engages the patient and ensures that she understands the condition and treatment options. A practitioner should also complete PPD assessment of some type at all prenatal and postpartum visits in order to assess response trends and catch changes in depressive symptoms. More research is needed to understand ways of overcoming the barriers identified in this review. There is also the need for more studies of high evidence level to be completed on the specific population of low-income women with a positive PPD screen.
